# Patient Characteristics Associated with Measurement of Routine Diabetes Care: An Observational Study

**DOI:** 10.1371/journal.pone.0121845

**Published:** 2015-03-30

**Authors:** Arna L. Van Doorn-Klomberg, Jozé C. C. Braspenning, Femke Atsma, Birgit Jansen, Margriet Bouma, René J. Wolters, Michel Wensing

**Affiliations:** 1 Scientific Institute for Quality of Healthcare, Radboud University Medical Center, Nijmegen, The Netherlands; 2 Dutch College of General Practitioners (NHG), Utrecht, The Netherlands; Providence VA Medical Center and Brown University, UNITED STATES

## Abstract

**Background:**

Non-modifiable patient characteristics, including age, gender, ethnicity as well as the occurrence of multi-morbidities, are associated with processes and outcomes of diabetes care. Information on these factors can be used in case mix adjustment of performance measures. However, the practical relevance of such adjustment is not clear. The aim of this study was to assess the strength of associations between patient factors and diabetes care processes and outcomes.

**Methods:**

We performed an observational study based on routinely collected data of 12,498 diabetes patients in 59 Dutch primary care practices. Data were collected on patient age, gender, whether the patient lived in a deprived area, body mass index and the co-occurrence of cardiovascular disease, chronic obstructive pulmonary disease, depression or anxiety. Outcomes included 6 dichotomous measures (3 process and 3 outcome related) regarding glycosylated hemoglobin, systolic blood pressure and low density lipoprotein-cholesterol. We performed separate hierarchical logistic mixed model regression models for each of the outcome measures.

**Results:**

Each of the process measure models showed moderate effect sizes, with pooled areas under the curve that varied between 0.66 and 0.76. The frequency of diabetes related consultations as a measure of patient compliance to treatment showed the strongest association with all process measures (odds ratios between 5.6 and 14.5). The effect sizes of the outcome measure models were considerably smaller than the process measure models, with pooled areas under the curve varying from 0.57 to 0.61.

**Conclusions:**

Several non-modifiable patient factors could be associated with processes and outcomes of diabetes care. However, associations were small. These results suggest that case-mix correction or stratification in assessing diabetes care has limited practical relevance.

## Introduction

Many quality programs for diabetes care aim at improving clinical processes and outcomes [1]. An important part of the goals set in these programs is the prevention of cardiovascular risk [2,3]. Relevant intermediate outcomes include glycated hemoglobin (HbA1c), blood pressure and serum cholesterol values. These outcome measures are associated with a range of non-modifiable patient characteristics, including gender, age, ethnicity and other socio-demographic factors [4–7]. For example, women in diabetes care have higher blood pressure and serum cholesterol levels compared to men [7]. In order to compare performance measures between practices, adjustment is suggested for these factors, as they could be unequally distributed in the different populations assessed [8]. Zaslavsky et al. [9] showed that adjustment for socio-demographic factors had little impact on most performance measures, but larger impact on a few measures, particularly when the measure involved a relatively large percentage of patients from a minority ethnic group. However, case mix adjustment is not consistently applied, possibly due to differential perspectives and uncertainties about when and how to adjust [10].

Besides socio-demographic factors, differences in the severity of the condition can be associated with diabetes control as well. The severity of diabetes mellitus can be defined as the co-occurrence of other conditions. In this article we focus on multi-morbidity, which can be defined as 'any co-occurrence of medical conditions within a person' [11]. The number of patients with multi-morbidity has risen enormously in the past decades [12]. A systematic review [13] showed that conditions that often occur in diabetes patients include cardiovascular diseases (CVD), depression and cancer. These conditions may be directly related to diabetes (e.g. CVD) or indirectly related (e.g. chronic obstructive pulmonary disease, COPD) [14]. Several studies have shown that the presence of different types of multi-morbid conditions (in several patient groups, including patients with diabetes) can lead to better cardiovascular risk control, both regarding processes and outcomes of care [15–17]. Multi-morbidity may lead to lowered patient compliance with treatment and advice [18,19]. Turner et al. [20] found that the relationship between multi-morbidity and healthcare outcomes may vary, depending on how closely related the conditions are. They suggest that related multi-morbid conditions may lead to improved care delivery, whereas unrelated multi-morbid conditions may have a negative impact on health outcomes [20]. Voorham et al. [21]specifically looked at the effects of multi-morbidity on medication treatment and found that diabetes-related multi-morbidity could induce treatment intensification for cardiovascular risk factors.

Information on multi-morbidity can be used in case mix adjustment of performance measures. However, the strength of the association between multi-morbidity factors, other case mix factors and measures of processes and outcomes of care is not clear. Therefore, the aim of this study was to (1) identify which patient factors and prevalent multi-morbid conditions are related to processes and outcomes of diabetes care, (2) how strong these associations are, and (3) the relative importance of the factors identified as predictors.

## Methods

The research was conducted under supervision of IQ healthcare, a scientific department focusing on quality improvement in healthcare, and as such forms part of the Radboud University Medical Centre in Nijmegen, the Netherlands. LINH is registered with the Dutch Data Protection Authority and data are handled according to the national data protection guidelines. The study was conducted in accordance with the Dutch Law for the Protection of Personal Data (Wet Bescherming Persoonsgegevens)[22] and the Declaration of Helsinki [23]. Based on the Dutch Law for the Protection of Personal Data no medical ethical committee approval was required for this study, therefore the medical ethical committee was not contacted. All data were anonymized before extraction and analysis.

### Data availability statement

Due to ethical restrictions, data are available from the Netherlands Information Network of General Practice (LINH). An English application form for the use of data can be found at http://www.nivel.nl/sites/default/files/bestanden/LINH-formulier_gegevensaanvraag_engels.rtf. Criteria for data access are displayed on this form. All data were anonymized before data extraction and analysis.

### Study design and population

We performed an observational study based on routinely collected data from Dutch primary care practices. In the Netherlands, routine diabetes care is primarily provided in general practice; therefore, in our study we focus solely on primary care. All data were collected as part of a national representative network of practices (LINH), in which data were extracted from the electronic patient records. In total, a sample of 12498 diabetes patients from 59 general practices was included in this study. All practices extracted their data in the year 2010. The extraction included information from all contacts with a time window of one year. This time period was based on the fact that all measures included in the study should be measured at least once a year according to the guidelines.

### Measures

We included demographic patient characteristics and information on prevalent multi-morbidities in the study. Data were collected on patient age, gender, whether the patient lived in a deprived area and body mass index (BMI). A systematic review showed that out of all conditions that are commonly treated in primary care, CVD, COPD, depression and anxiety most often co-occur with diabetes [13]. Information on multi-morbidity was based on the ICPC codes [24], and included as a dichotomous variable for each condition. CVD included ischaemic heart disease with or without angina, acute myocardial infarction, heart failure, atrial fibrillation, hypertension, transient cerebral ischaemia, cerebrovascular disease, stroke and atherosclerosis. Anxiety and depression included the ICPC codes for feeling depressed, anxiety disorder or anxiety state and depressive disorder. Furthermore, the number of diabetes related consultations per year was included. Whenever the ICPC code for diabetes was recorded in the medical record, the consultation was recorded as diabetes related. Consultations can be provided by the general practitioner or other staff in the general practice, including a nurse practitioner. In this study, we only included the frequency of the consultations; we did not have information on the caregiver that provided the consultation. We also recorded whether patients received a prescription of lipid lowering medication, anti-diabetic medicine (oral, insulin or both) or blood pressure lowering medication.

Outcome measures included 6 dichotomous measures regarding glycosylated hemoglobin (HbA1c), systolic blood pressure (BP) and low density lipoprotein-cholesterol (LDL-C). We included whether the target level (as set in the Dutch guideline [25]) was measured during consultation (yes/no) and, for those cases with a valid measurement, whether it was achieved (yes/no). The most recent valid data for each patient were included in the dataset.

### Analyses

Descriptive statistics were used to describe the patient and practice characteristics. A missing data analysis was performed since length and weight (to calculate BMI) were not recorded structurally each year. We performed multiple imputation to complete missing values on BMI data, using 5 imputation sets. A larger number of imputation sets has been recommended. However, since our dataset consisted of a large number of patients, the difficulties of applying a large number of imputation sets (>10) in a reasonable amount of time outweighed the benefits. Practices, main outcomes and predictors were included in de imputation models. We also included practice in the imputation models to account for clustering effects.

To assess which patient factors were associated with clinical processes and outcomes of diabetes care, logistic mixed models were performed on the imputation models in order to obtain pooled results, taking into account the clustering of patients within practices. We ran six separate hierarchical logistic regression models, one for each of the outcome measures (HbA1c, BP and LDL-C measured, HbA1c, BP and LDL-C within target level). Target levels were set at 53 mmol/mol, 140 mm Hg and 2.5 mmol/l respectively. In each model, we included patient age, gender, number of diabetes related consults (above or below median), whether the patient lives in deprived area, BMI and presence of ICPC code related to CVD, COPD and / or depression and anxiety (dichotomous measures). We assessed the discriminative power of the model by calculating the area under the curve in the imputation sets (pooled AUC) and we also calculated pooled fixed effects (OR). In order to assess whether specific factors can be left out from the model without decreasing the effect size, we then reduced each of the 6 models in accordance with the maximum log-likelihood test (p<0.10) and again calculated pooled fixed effects and pooled AUC's. The multiple imputation and logistic multilevel models were performed in R version 3.03. Descriptive statistics were performed in SPSS version 20.

## Results

### Study population and measures

11,718 out of 12,498 patients from 59 practices were included in the analyses of process measures. 689 patients were excluded because they were in hospital care instead of primary care, 91 patients were excluded due to age (younger than 18 years). This population is a reasonable representation of all Dutch diabetes patients in primary care Table 1. Subsets of 8028, 8936 and 9257 patients were included in the analysis of outcome measures on LDL cholesterol, HbA1c and systolic blood pressure respectively. The percentage of patients with outcomes below target level was slightly lower than the percentages found in a Dutch multidisciplinary care study; however, in the latter, practices could exclude particular patient groups that are more difficult to treat. Also, the study population scored substantially lower on the registration of the patient outcomes. In Table 2, we report the variation on the process measures across the 59 practices. We found that for each of the process measures, the variation between practices is sufficiently large to show possible effects of patient determinants.

In our missing data analysis we found that as expected the main measure with missing data was BMI (3436 missing, 29.3%). Other variables with missing data included age (1 missing, 0.01%) and whether the patient lives in a deprived area (81 missing, 0.69%). Results from a MCAR test showed that the missing data were not completely at random.

**Table 1:** Patient characteristics and overall scores on indicators.

patient characteristics	study population	Dutch diabetes population^^^ ^,^ ^A^ ^,^ ^B^ ^,^ ^D^
N	11,718	834,100
Women	49.8%	50.1%
mean age (SD)	65.8 (13.3)	67.7 (12.6)
mean Body Mass Index (SD)	29.6 (5.3)	-
patients with funds for deprived areas	5.9%	-
mean number of consultations per year (SD)	7.6 (6.9)	-
mean number of DM related consultations per year (SD)	2.8 (3.4)	-
***multi-morbid conditions***		
a) cvd	69.8%	-
b) COPD	8.5%	8.4%
c) depression and anxiety	13.2%	17.6%
***medication prescriptions***		
antidiabetic medicine		
a) no medication	31.3%	22%
b) oral medication only	52.3%	62%
c) oral medication and insulin	10.4%	10%
d) insulin only	6.0%	4%
blood pressure lowering medication	69.3%	-
cholesterol lowering medication	64.0%	67.0%
indicator outcomes	study population	Dutch studymultidisciplinary care^C^
HbA1c value measured	76.7%	91.4%
mean HbA1c value (SD)	50.8 (11.8)	-
HbA1c value below target level (53)	67.3%	68.8%
systolic blood pressure measured	79.0%	89.9%
mean systolic blood pressure value (SD)	140.3 (18.1)	-
systolic blood pressure below target level (140)	49.1%	52.3%
LDL cholesterol level measured	69.4%	85.3%
mean LDL cholesterol level (SD)	2.59 (.93)	-
LDL cholesterol level below target level (2.5)	49.6%	53.1%

source data on the Dutch population:

A. RIVM. National Compass Population Health (Nationaal Kompas Volksgezondheid). 2011 [6–19–14]; Available from: http://www.nationaalkompas.nl/gezondheid-en-ziekte/ziekten-en-aandoeningen/endocriene-voedings-en-stofwisselingsziekten-en-immuniteitsstoornissen/diabetes-mellitus/omvang/.

B. Ubink-Veltmaat LJ, Bilo HJ, Groenier KH, Houweling ST, Rischen RO, Meyboom-de Jong B. Prevalence, incidence and mortality of type 2 diabetes mellitus revisited: a prospective population-based study in The Netherlands (ZODIAC-1). European journal of epidemiology. 2003;18(8):793–800. Epub 2003/09/17.

C. Transparancy in multidisciplinary care. In Dutch: Transparantie ketenzorg Diabetes Mellitus. Rapportage zorggroepen 2010. Utrecht, The Netherlands: Dutch National Organization for multidisciplinary care, 2012.

D. van Doorn-Klomberg A, Braspenning J, Bouma M. Report Dutch Practice Accreditation. Medical care in 2012. In Dutch: Rapportage NHG-Praktijkaccreditering: gegevens over het medisch handelen in 2012. Nijmegen, The Netherlands: Radboud University Medical Center, IQ healthcare, 2013.

^ Several characteristics of our study population could not be contrasted with the Dutch population or other comparable study populations because no suitable data could be found.

**Table 2:** Variations in clustered scores (per practice) on process indicators.

indicator outcomes		**study population**
HbA1c value measured	mean	76.0%
	SD (range)	10.2% (50.8–96.7)
systolic blood pressure measured	mean	76.9%
	SD (range)	15.0% (7.0–96.9)
LDL cholesterol level measured	mean	67.0%
	SD (range)	18.3% (2.0–93.1)

### Multilevel logistic regression analyses process measures

Each of the process measure models showed moderate effect sizes, with pooled areas under the curve (AUCs) that varied between 0.66 and 0.76. The closer the AUC value approaches 1, the better the model can distinguish whether processes are performed or not based on the factors included in the model. An AUC of 0.5 would indicate that the model does not perform better than chance. A pooled AUC of. 76 indicates that for each pair of patients (one with processes performed and one without processes performed), the model could classify these correctly in 76% of the cases. The effect sizes could be maintained with a reduced model. In the complete models, HbA1c, systolic blood pressure and LDL cholesterol values were measured more often in older patients, see Table 3. Furthermore, patients with CVD more often had their outcomes measured, with the largest odds for measurement of blood pressure (OR 1.75, 95% CI 1.56–1.98). In other words, as a diabetes patient with CVD as multi-morbidity, the odds of having your blood pressure measured were 1.75 times higher than for diabetes patients without CVD. On the other hand, patients with COPD had a lower probability to have their blood pressure measured (OR 0.72, 95% CI 0.59–0.87). COPD patients also had a smaller probability of HbA1c and LDL cholesterol measurements compared to patients without COPD. For each of the three process measures there was an association of the frequency of diabetes related consultations and the probability of the value being measured (ORs vary between 5.6 and 14.5). In fact, this factor on its own explained a large part of the effects found in the models (pooled AUC between 0.66 and 0.71) compared to models that included all factors (pooled AUC between 0.70 and 0.76).

**Table 3:** Factors associated with indicator processes: measure recorded within study period.

	**complete model**		**reduced model**		**DM consultation only model**	
Factor^#^	OR	95% CI	OR	95% CI	OR	95% CI
**hbA1c**						
age in years	1.03	1.03–1.03	1.03	1.02–1.03	-	-
gender male	1.01	0.92–1.12	-	-	-	-
patient in deprived area	1.03	0.78–1.36	-	-	-	-
cvd	1.15	1.03–1.29	1.17	1.05–1.31	-	-
depression and anxiety	1.08	0.93–1.25	-	-	-	-
COPD	0.81	0.67–0.97	0.81	0.68–0.97	-	-
body mass index in kg/m^2^	1.01	1.00–1.03	-	-	-	-
DM consultation count above median	14.4	12.3–16.7	14.4	12.3–16.8	15.4	13.2–17.9
*AUC*	*0*.*76*		*0*.*76*		*0*.*71*	
*N*	*11718*		*11718*		*11718*	
**systolic blood pressure**						
age in years	1.03	1.03–1.04	1.03	1.03–1.04	-	-
gender male	1.04	0.93–1.16	-	-	-	-
patient in deprived area	1.11	0.83–1.49	-	-	-	-
cvd	1.75	1.56–1.98	1.77	1.57–1.99	-	-
depression and anxiety	0.93	0.80–1.09	-	-	-	-
COPD	0.72	0.59–0.87	0.72	0.59–0.87	-	-
body mass index in kg/m^2^	1.01	0.99–1.02	-	-	-	-
DM consultation count above median	14.5	12.3–17.1	14.5	12.3–17.1	15.5	13.1–18.2
*AUC*	*0*.*77*		*0*.*77*		*0*.*71*	
*N*	*11718*		*11718*		*11718*	
**LDL cholesterol**						
age in years	1.02	1.01–1.02	1.02	1.01–1.02	-	-
gender male	1.17	1.06–1.29	1.16	1.06–1.27	-	-
patient in deprived area	0.87	0.66–1.13	-	-	-	-
cvd	1.31	1.18–1.46	1.32	1.19–1.47	-	-
depression and anxiety	1.03	0.90–1.18	-	-	-	-
COPD	0.81	0.69–0.95	0.81	0.69–0.95	-	-
body mass index in kg/m^2^	1.00	0.99–1.02	-	-	-	-
DM consultation count above median	5.60	4.99–6.28	5.61	5.00–6.30	5.92	5.28–6.63
*AUC*	*0*.*70*		*0*.*69*		*0*.*66*	
*N*	*11718*		*11718*		*11718*	

# reference categories: female, less than median diabetes related contacts per year, without funds for deprived area, no multi-morbidities.

### Multilevel logistic regression analyses outcome measures

The effect sizes of the outcome measure models were considerably smaller than the process measure models, with pooled AUCs varying from 0.57 to 0.61. This indicates that the model did not perform much better than chance. The probability of achieving the systolic blood pressure target level was higher in older patients, while the probability of an LDL cholesterol level within target was lower in older patients Table 4. Gender was also associated with outcomes, with lower probabilities of good HbA1c and blood pressure control and a higher probability of good LDL cholesterol control in male patients compared to female patients. Patients with CVD had a higher probability that the HbA1c level was within target level and a lower probability that the blood pressure was within target level. Patients with depression and anxiety also had a higher probability of achieving an HbA1c level within target level. Furthermore, these patients had a lower probability of achieving good LDL cholesterol control. Blood pressure and HbA1c levels were less often below target level in patients with a higher BMI. A relatively high number of diabetes consultations was associated with poorer HbA1c control and better LDL cholesterol control.

**Table 4:** Factors associated with indicator outcomes: outcome within target level.

	**complete model**		**reduced model**	
Factor^#^	OR	95% CI	OR	95% CI
**hbA1c**				
age in years	1.00	1.00–1.01	1.01	1.00–1.01
gender male	0.83	0.75–0.91	0.83	0.75–0.91
patient in deprived area	0.84	0.65–1.07	-	-
cvd	1.10	0.99–1.23	-	-
depression and anxiety	1.19	1.03–1.36	1.18	1.03–1.36
COPD	0.95	0.81–1.12	-	-
body mass index in kg/m^2^	0.97	0.96–0.98	0.97	0.96–0.98
DM consultation count above median	0.54	0.48–0.60	0.54	0.48–0.60
*AUC*	*0*.*59*		*0*.*58*	
*N*	*8936*		*8936*	
**systolic blood pressure**				
age in years	0.97	0.97–0.98	0.97	0.97–0.98
gender male	0.92	0.84–1.00	0.92	0.84–1.00
patient in deprived area	0.92	0.71–1.18	-	-
cvd	0.54	0.49–0.60	0.54	0.49–0.60
depression and anxiety	1.07	0.94–1.21	-	-
COPD	1.13	0.97–1.31	-	-
body mass index in kg/m^2^	0.97	0.96–0.98	0.97	0.96–0.98
DM consultation count above median	1.04	0.94–1.16	-	-
*AUC*	*0*.*61*		*0*.*61*	
*N*	*9257*		*9257*	
**LDL cholesterol**				
age in years	1.01	1.01–1.02	1.01	1.01–1.01
gender male	1.47	1.34–1.62	1.45	1.33–1.59
patient in deprived area	1.44	1.09–1.89	1.44	1.10–1.90
cvd	1.19	1.07–1.32	1.20	1.08–1.34
depression and anxiety	0.84	0.74–0.96	0.84	0.74–0.96
COPD	1.03	0.87–1.21	-	-
body mass index in kg/m^2^	1.01	1.00–1.02	-	-
DM consultation count above median	1.33	1.19–1.48	1.33	1.19–1.48
*AUC*	*0*.*57*		*0*.*57*	
*N*	*8028*		*8028*	

# reference categories: female, less than median diabetes related contacts per year, without funds for deprived area, no multi-morbidities.

## Discussion

In this study the associations between several non-modifiable patient characteristics and prevalent multi-morbidities with processes and outcomes of diabetes care in Dutch general practices were explored. We found that the models on processes of care showed moderate effects, suggesting that patient factors had some impact on diabetes care indicators. On the other hand, effects on outcome measures were small. Measurement of the number of diabetes consultations, as a measure of patient health service utilization, was the main contributor to the effects found.

### Explanation of findings

Factors incorporated in our study only had small to moderate pooled effects on processes and outcomes of care. This is in line with a study by Marceau et al. [26], who reported that from the 21.1% of variance in diabetes management that could be explained by patient, provider and organizational factors, only 2.2% could be explained by patient factors. The main contributor was the diabetes consultation frequency. Since in general all patients receive automated invitations for their 3-monthly and yearly consultations, this measure reflects patients’ attitudes and compliance. The effects found are in line with other literature that shows that poor adherence to treatment plans (such as failure to come to an appointment) can have negative effects on processes and outcomes of care [27].

Since effect sizes found are moderate to small, it may be debated whether to take these case mix factors into account in the calculation of scores on population level, either through correction or stratification. The small changes in scores may not weigh up against the troubles of data collection and increased complexity of score interpretation. Furthermore, Nicholl [28] suggests that correction can actually increase the bias in measurements, especially when interactions between factors are not taken into account properly. However, it is possible that other patient factors than in this study are relevant for diabetes care processes and outcomes, or that the examined factors are relevant for health outcomes that were not examined in this study. Also, for the evaluation of individual patients it is important to be aware of the possible risk factors, since they may have large influence in a single patient. Furthermore, when the total number of diabetes patients in a practice is relatively small, one should account for a larger variation in the score [29]. In such case, we suggest that accounting for a larger error margin is better than correcting for multiple factors, since this will only increase the bias due to the lower precision of the data.

Results on measurement of outcomes are in line with previous literature that shows that the influence of a multi-morbidity depends on whether the conditions are strongly related to each other [20]. We found that in patients with COPD, a condition that is less strongly related to diabetes, processes of care were less often according to the guidelines. It is possible that during consultations with COPD patients, priority is given to address those issues that cause the highest burden on the patient quality of life the most. These issues are more likely to be COPD related rather than diabetes related. On the other hand, patients with a strongly related condition, CVD, were more likely to have their blood pressure measured. Results are similar to a Dutch study by Nouwens et al., in which better cardiovascular risk treatment in diabetes patients but not in COPD patients was reported [16]. Our findings are also in line with the study by Voorham et al. [21]. They looked at the association between multi-morbidity and treatment intensification. In their study, new occurrences of diabetes-related multi-morbidities such as cardiovascular disease were related with better risk factor treatment, whereas no effects were found for non-diabetes related multi-morbidities.

Our results show that patients with depression and anxiety have a higher probability to have good HbA1c control than others, whereas the probability is lowered that they have good cholesterol control. Although literature shows that depression is associated with poorer medication adherence [30], evidence regarding the effects on HbA1c, blood pressure and cholesterol control is inconclusive [31,32]. Possibly patients with anxiety are more sensitive for side-effects of lipid lowering medication, resulting in poor cholesterol control.

### Strengths and limitations

In this observational study, there were several factors associated with diabetes care that we could not take into account. These include hereditary factors and lifestyle factors, which have known associations with development of diabetes [33]. We also did not include duration and severity of the illness in our study, which is an important limitation. Furthermore, medication compliance may have varied between patients, which may have also influenced the scores. Other studies show that longer diabetes duration is associated with more diabetes complications such as retinopathy [34] and coronary heart disease [35]. These factors may explain part of the variation that we could not account for with our model. Poor outcomes of care may be accounted for by factors that we did not include in our dataset, including presence of pancreatic illnesses or issues with medication tolerance. However, these factors only regard small parts of the population of diabetic patients seen by the general practitioner. Therefore, we expect that this has not had a large influence on our study.

Our study was performed on a large dataset that formed an adequate reflection of the diabetes patients treated in primary care. There were several limitations to the use of this dataset. We could only analyze the outcomes of care for those patients that had a measurement of the outcome, i.e. scored positively on the process measure. This can cause a bias in our study. It may be the case that practitioners are more likely to perform measurements when they expect that patients need treatment; leading to a slightly lower percentage of patients with a measurement within target level compared to those without a measurement. On the other hand, there may have been an underrepresentation of patients that show higher noncompliance to treatment. It is to be expected that outcomes are not within target level for this group of patients, which may cause a bias in the opposite direction. However, we argue that this bias also occurs in routine care. Since our aim was to develop a model of determinants that approaches everyday care, the large study population that we used was valid for its purpose.

Since data were collected through an automated extraction of medical records, it is likely that there is an effect of poor registration. This is reflected by the large difference in measurement of outcomes in our study population compared to the report on multidisciplinary care. In the latter study, the practice collected data from their registration themselves, which meant that they could retrieve misplaced information more easily. The registration bias in our study may have led to a bias in our study population. Although poor registration may have led to lower scores on processes of care, in general our population was reasonably well controlled. This may have decreased the likelihood of finding associations in this study.

### Conclusion

Several non-modifiable patient factors could be associated with processes and outcomes of diabetes care. The main contributor to improved processes of care was the frequency of diabetes consultations. However, especially regarding the outcomes of care, associations were small. Therefore, our results do not support the need for case-mix correction or stratification suggested in previous literature. Accounting for a certain margin of error around the scores without further correction may be more beneficial for users in practice.
